# Role of Damage-Associated Molecular Patterns (DAMPs/Alarmins) in Severe Ocular Allergic Diseases

**DOI:** 10.3390/cells11061051

**Published:** 2022-03-20

**Authors:** Ken Fukuda, Waka Ishida, Tatsuma Kishimoto, Isana Nakajima, Yusaku Miura, Tamaki Sumi, Kenji Yamashiro

**Affiliations:** Department of Ophthalmology and Visual Science, Kochi Medical School, Kochi University, Kochi 783-8505, Japan; wakai@kochi-u.ac.jp (W.I.); t.kishimoto@kochi-u.ac.jp (T.K.); jm-i-nakajima@kochi-u.ac.jp (I.N.); miurasaku@kochi-u.ac.jp (Y.M.); sumitama@kochi-u.ac.jp (T.S.); yamashk@kochi-u.ac.jp (K.Y.)

**Keywords:** alarmins, allergy, atopic keratoconjunctivitis, vernal keratoconjunctivitis, interleukin, cornea, conjunctiva, corneal epithelial cells, eosinophils, corneal fibroblasts

## Abstract

Severe ocular allergic diseases, such as atopic keratoconjunctivitis and vernal keratoconjunctivitis, cause severe allergic inflammation in the conjunctiva and corneal epithelial damage, resulting in visual disturbances. The involvement of damage (danger)-associated molecular patterns (DAMPs/alarmins) in the pathogenesis of these diseases has been recognized. Alarmins released from damaged corneal epithelial cells or eosinophils play a critical role in the induction of corneal lesions, vicious loop of corneal injury, and exacerbation of conjunctival allergic inflammation. Alarmins in the conjunctiva also play an essential role in the development of both allergic inflammation, based on the acquired immune system, and type 2 inflammation by innate immune responses in the ocular surface. Therefore, alarmins may be a potentially important therapeutic target in severe refractory ocular allergic diseases.

## 1. Introduction

**Figure 1:**
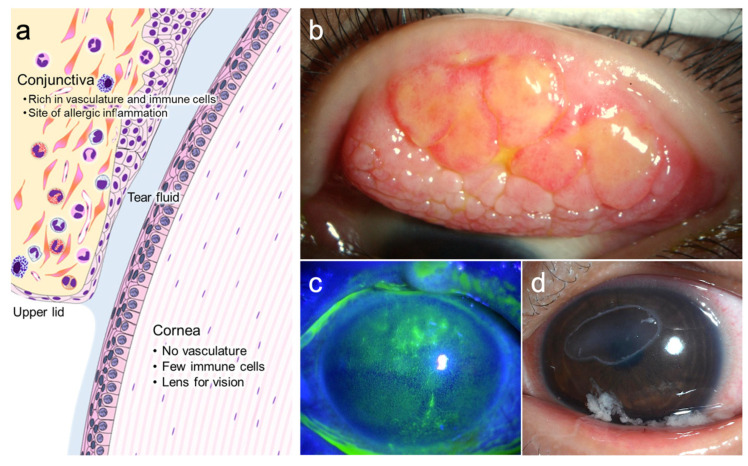
Anatomy of the ocular surface and clinical pictures of vernal keratoconjunctivitis (VKC). The conjunctiva and cornea lie adjacent to each other with the tear fluid (**a**). In patients with VKC, giant papillae at the upper tarsal conjunctiva (**b**) and corneal epithelial damage, such as exfoliated superficial punctate epithelial keratitis (**c**) or shield ulcer (**d**), are observed.

Allergic diseases, such as asthma, pollinosis, rhinitis, atopic dermatitis, and conjunctivitis, are among the most common diseases known worldwide [1]. The incidence of ocular allergic diseases is especially high; 48.3% of the Japanese population is estimated to suffer from ocular allergic diseases [2]. The ocular surface is formed from the tissues adjacent to the conjunctiva and cornea, which have distinct immunological and optical roles (Figure 1). The conjunctiva, which is a mucosal tissue rich in blood vessels and immune cells, is not involved in vision. On the other hand, the cornea is a transparent tissue acting as a lens. The cornea is an avascular tissue, and few immune cells reside in the central part of the cornea. The nature of the conjunctiva and corneal epithelium is also different. The conjunctival epithelium contains goblet cells, which produce mucin and have a relatively weak barrier function; hence, allergens can easily penetrate the epithelium. The corneal epithelium, on the other hand, has a potent epithelial barrier that prevents the entry of microorganisms or allergens and has few immune cells, making it less prone to inflammation and allergic reactions. However, severe allergic inflammation in the conjunctiva often causes corneal injury [3], and alarmins/damage (danger)-associated molecular patterns (DAMPs) are involved in the pathogenesis of these corneal lesions. In this review, we discuss the role of alarmins/DAMPs in ocular allergic diseases.

## 2. Corneal Involvements in Severe Ocular Allergic Diseases

Allergic conjunctival diseases are classified into four categories: allergic conjunctivitis, atopic keratoconjunctivitis (AKC), vernal keratoconjunctivitis (VKC), and giant papillary conjunctivitis (GPC) [4]. Seasonal (perennial) allergic conjunctivitis is caused by a type I allergic reaction following sensitization to allergens, such as pollen or house dust and re-exposure to these allergens. Giant papillary conjunctivitis is characterized by proliferative changes in the conjunctiva owing to mechanical irritation attributed to contact lens usage or surgical sutures. AKC is a chronic keratoconjunctivitis associated with atopic dermatitis of the face, while VKC is associated with proliferative changes in the conjunctiva, eosinophilic inflammatory cell infiltration, and various types of corneal epithelial lesions. Allergic conjunctivitis is mainly caused by the acquired immune system, while GPC is mainly caused by the innate immune system. Both innate and acquired immunities are involved in the pathophysiology of AKC and VKC [5,6,7,8]. In recent years, the concept of “type 2 immune reaction,” which includes innate immune responses, has been widely accepted in the understanding of allergic diseases [9]. In type 2 immune reactions, epithelial cells function as a biological barrier to release type 2 immune response-initiating cytokines, such as IL-33, thymic stromal lymphopoietin (TSLP), and IL-25 upon exposure to allergens. These epithelial cell-derived cytokines, also known as alarmins, trigger the activation of both innate and acquired immune responses, resulting in allergic inflammation. The epithelium of the ocular surface also expresses these cytokines and is thought to play an important role, especially in AKC and VKC [10,11,12].

Although the primary allergic reaction occurs in the conjunctiva, severe allergic inflammation in the conjunctival giant papillae (Figure 1b) often results in corneal damage, leading to vision loss in AKC and VKC. Therefore, these two diseases are referred to as severe ocular allergic diseases. Corneal lesions include superficial punctate keratitis, erosion, shield ulcers, and corneal plaques, which can cause visual impairment and eye pain (Figure 1c,d). Prolonged corneal lesions may result in scarring of the corneal stroma and vascular invasion, resulting in permanent vision loss [3].

**Figure 2:**
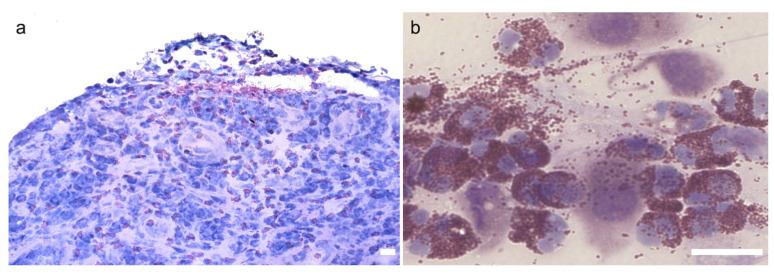
Eosinophils in giant papillae and tear fluids in patients with vernal keratoconjunctivitis (VKC). Accumulation of eosinophils (stained in red with Hansel staining) are apparent under a conjunctival epithelial erosion in giant papillae (**a**). Numerous degranulated eosinophils with detached epithelial cells were observed in the ocular discharge (**b**). Scale bars: 20 μm. Reprinted with permission from [3]. Copyright 2022 Japanese Society of Allergology.

Many eosinophils and eosinophil granule proteins are detected in the conjunctiva and tear fluid of patients with VKC/AKC (Figure 2) [13,14,15], and cytotoxic proteins derived from eosinophils are deposited in the corneal ulcers [16,17], suggesting that corneal lesions in these diseases are mainly caused by eosinophils. Furthermore, the severity of corneal diseases in AKC reportedly correlates with the concentration of eosinophils and eotaxin-1 (CCL11) in the tear fluid [18]; therefore, CCL11 is thought to be an important chemokine that allows eosinophils to migrate to the cornea. We and others investigated the cells producing CCL11 in the tear fluid using cultured corneal epithelial cells and stromal keratocytes (fibroblasts) and found that corneal fibroblasts produce large amounts of CCL11 owing to the synergistic effects of type 2 cytokines (IL-4, IL-13) and tumor necrosis factor-α (TNF-α) [19,20]. Similarly, vascular cell adhesion molecule-1 (VCAM-1), an adhesion molecule for eosinophils, was found to be expressed by the synergistic action of type 2 cytokines (IL-4 and IL-13) and TNF-α in corneal fibroblasts but not in corneal epithelial cells [21,22]. Although epithelial cells and fibroblasts are structural cells distributed throughout the body, their properties are not uniform, and the chemokine production capacity of these cells in the target tissues of allergic diseases also varies in different tissues. For example, epithelial cells in the lungs, epithelial cells and fibroblasts in the skin, and fibroblasts in the cornea are responsible for the production of CCL17 (thymus and activation-regulated chemokine) [23,24]. 

Until recently, corneal epithelium was thought to be merely damaged by eosinophils and to have no role in the pathogenesis of corneal lesions secondary to ocular allergic disease. However, Matzinger proposed the danger theory, which states that cellular and tissue damage triggers inflammation and that the substances that cause endogenous danger signals are collectively termed DAMPs/alarmins and trigger inflammatory responses [25]. The involvement of these damaged cells in ocular allergic inflammation has also been investigated. 

## 3. The Role of Alarmins/DAMPs in the Pathogenesis of Corneal Lesions in AKC/VKC

DAMPs/alarmins include cytokines, such as IL-1α and IL-33, high-mobility group box (HMGB), S100 protein, heat shock protein, nucleosomes, defensin, cathelicidin, and many other substances [25,26]. Excessive release of alarmins has been shown to potently stimulate the immune system and induce allergic inflammation in asthma and atopic dermatitis [27,28,29]. In this section, we review the role of these alarmins in allergic inflammation of the ocular surface.

### 3.1. The Roles of Alarmins from Damaged Corneal Epithelial Cells

**Figure 3:**
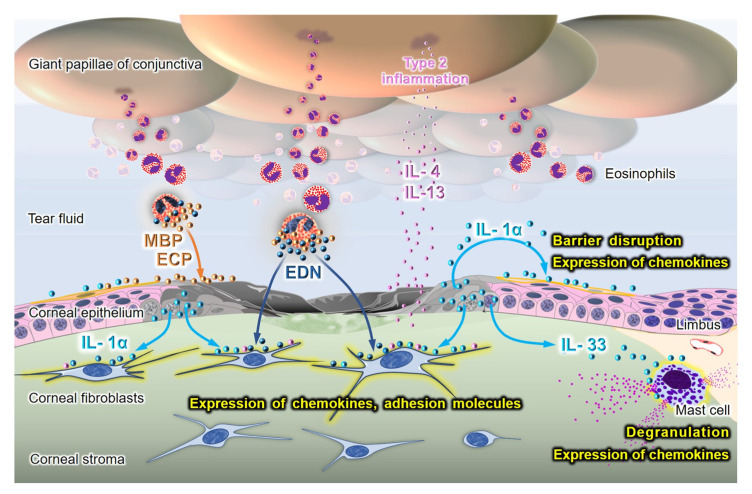
Involvement of alarmins in the pathogenesis of corneal lesions in severe ocular allergic diseases. Alarmins from damaged epithelial cells and eosinophils exacerbate the allergic inflammation and corneal lesions through barrier disruption and recruitment of eosinophils. IL; interleukin, EDN; eosinophil-derived neurotoxin, MBP; major basic protein, ECP; eosinophil cationic protein.

We and others have investigated the effect of alarmins from necrotic corneal epithelial cells on the ocular surface in vitro [30,31,32]. The supernatant of the necrotic corneal epithelial cells activated intracellular nuclear factor-κB and promoted the production of inflammatory cytokines, such as IL-8 and IL-6, by healthy corneal epithelial cells. In addition, alarmins decrease the barrier function of these cells (Figure 3) [31].

The effects of alarmins released from damaged epithelial cells on corneal fibroblasts have also been investigated. The addition of the supernatant from necrotic epithelial cells to corneal fibroblasts enhanced the production of various mediators, including IL-6, MCP-1 (CCL-2), RANTES (CCL-5), MCP-2 (CCL8), eotaxin-1 (CCL11), and IL-8 (CXCL8) [30,32]. Furthermore, the production of CCL11 was synergistically promoted by the addition of necrotic supernatant in the presence of IL-4 or IL-13 [32]. In addition to chemokines, an adhesion molecule to eosinophils, VCAM-1, was synergistically promoted by the co-stimulation of the necrotic epithelial supernatant with IL-4 or IL-13 (Figure 3) [32]. 

These inflammatory reactions by both corneal epithelial cells and fibroblasts in response to alarmins were inhibited by IL-1 receptor antagonists or anti-IL-1α antibodies, indicating that IL-1α released from necrotic corneal epithelial cells plays a critical role [31,32]. Alarmins from damaged epithelial cells contribute to the exacerbation of eosinophilic inflammation in patients with AKC/VKC via the epithelial barrier dysfunction and chemokine expression of fibroblasts. 

### 3.2. The Role of Alarmins in Eosinophils

Eosinophils and eosinophil granule-derived secretory proteins, such as eosinophil major basic protein (MBP), eosinophil cationic proteins (ECP), and eosinophil-derived neurotoxin (EDN), are increased in the conjunctiva and tears of patients with VKC/AKC [33,34]. The involvement of eosinophils in corneal lesions in VKC/AKC is also associated with alarmins/DAMPs. MBP and ECP have cytotoxic properties in various cells, including corneal epithelial cells [17,35,36,37]. MBP was observed to cover corneal epithelial defect lesions [16]. Therefore, these proteins directly induce corneal epithelial cell death in patients with VKC/AKC and indirectly enhance allergic inflammation through the release of alarmins by dying epithelial cells. Second, EDN reportedly acts as an alarmin in allergic inflammation [38]. EDN is an endogenous ligand of toll-like receptor (TLR) 2. EDN activates dendritic cells and enhances type 2 immune responses through TLR2- myeloid differentiation factor 88 signaling pathway [38]. Both corneal epithelial cells and fibroblasts express functional TLR2 [39,40]. Activation of TLR2 by peptidoglycan derived from *Staphylococcus aureus*, combined with IL-4/IL-13, reportedly synergistically induce CCL11 and VCAM-1 expression in these cells [41]. Although the direct effect of EDN on the activation of TLR2 in corneal cells has not been examined, EDN may act on TLR2 and induce CCL11 and VCAM-1 expression in corneal fibroblasts (Figure 3). Thus, EDN from eosinophils in collaboration with IL-4/IL-13 may be involved in the exacerbation of eosinophil accumulation in the cornea, as well as in the enhancement of antigen-specific type 2 immune responses in the conjunctiva.

### 3.3. The Role of Alarmins in Animal Models of Ocular Allergy

#### 3.3.1. Corneal Epithelium Debridement Model

Although corneal injury occurs in AKC/VKC, there are few reports of corneal involvement in animal models of ocular allergic disease. Mechanical injury of the corneal epithelium in rabbits induced the release of alarmin IL-1α [42] and local inhibition of IL-1α by a topical IL-1 receptor antagonist downregulated inflammatory cell infiltration into the cornea following corneal epithelial scraping [43]. These studies suggest the importance of IL-1α as an alarmin in vivo in sterile inflammation induced by corneal epithelium injury, consistent with the in vitro results [31].

We investigated the role of corneal epithelial injury in ocular allergic inflammation by mechanically debriding the corneal epithelium in rats [44]. Sensitized or non-sensitized rats were administered eye drops of ragweed pollen after the corneal epithelium was abraded, and the rate of wound healing was examined. Corneal epithelial wound healing was significantly delayed in sensitized rats compared to non-sensitized rats following the administration of ragweed pollen eye drops. This result suggests that inflammatory cells, such as eosinophils, and mediators released during conjunctival allergic inflammation inhibit corneal epithelial wound healing, as observed in patients with AKC/VKC. In addition, we compared the degree of conjunctival inflammation following pollen drop administration in sensitized rats with and without corneal epithelial abrasion. The number of infiltrated eosinophils in the conjunctiva significantly increased in the group in which the corneal epithelium was removed. This result demonstrates an interaction between the conjunctiva and cornea, in which debridement of the corneal epithelium conversely exacerbates allergic inflammation in the conjunctiva [44,45].

#### 3.3.2. Mast Cells as Sensors of Alarmins

In addition to their critical role in type 1 allergic reactions by the acquired immune system, mast cells have recently been identified as sensors of cell injury by the recognition of alarmins [46]. The role of mast cells in the corneal limbus as sensors has been investigated on the ocular surface [47,48]. When the cornea is injured, limbal mast cells sense IL-33 derived from the injured corneal epithelium, degranulate, and produce chemokines, resulting in the infiltration of inflammatory cells into the cornea. Topical neutralization of IL-33 suppresses mast cell activation and subsequent inflammatory responses in the cornea [47]. As IL-33 is highly expressed in the conjunctival epithelium of giant papillae in patients with AKC [12], damaged conjunctival epithelium-derived IL-33 may also act on conjunctival mast cells.

#### 3.3.3. Role of IL-33 in Ocular Allergy Induced by Acquired and Innate Immune Systems

In addition to the corneal injury model, the role of IL-33 in ocular allergic diseases has been clarified in several studies using mouse models of allergic conjunctivitis. Epithelial cell-derived cytokines, including IL-33, TSLP, and IL-25, are recognized as alarmins, as well as type 2 immune response-initiating cytokines. Matsuba et al. reported that the simultaneous application of ragweed pollen and IL-33 as eye drops in sensitized mice significantly exacerbated the clinical symptoms and eosinophil infiltration of the conjunctiva [49]. These results are consistent with the results of in vitro experiments demonstrating the synergistically promoted mast cell degranulation by the co-stimulation of antigens and IL-33 in cultured mast cells [50]. Asada et al. also demonstrated a significant suppression of clinical symptoms, number of infiltrated eosinophils in the conjunctiva, and expression of type 2 inflammatory cytokines in IL-33-deficient mice [11]. These studies revealed that IL-33 is essential for the development of pollen-induced allergic conjunctivitis, which depends on the acquired immune system. Sugita et al. also established a model of conjunctivitis induced by the innate immune system in mice and demonstrated that the inflammatory response was markedly attenuated in IL-33-deficient mice [51]. Li et al. also revealed the involvement of IL-33 in pollen/TLR4 innate immunity; short ragweed pollen stimulated IL-33/ST2 signaling that triggers Th2-dominant allergic inflammation in the ocular surface via the TLR4/Myd88 pathway in mice [52]. These results indicate that IL-33 also plays an essential role in type 2 inflammation caused by innate immune responses on the ocular surface.

Furthermore, transgenic mice in which IL-33 is highly expressed in the epithelium of skin, conjunctiva, and cornea using the keratin 14 promoter reportedly demonstrate severe inflammation in the conjunctiva, as well as corneal epithelial defects and lesions resembling corneal plaques seen in AKC/VKC [53]. This study suggests that excessive expression of IL-33, one of the alarmins in the epithelium, adequately induces ocular surface inflammation and manifests clinical conditions similar to AKC/VKC, and that alarmins play a critical role in the pathogenesis of AKC/VKC.

#### 3.3.4. Role of TSLP and IL-25 in Ocular Allergy Induced by Acquired and Innate Immune Systems

In addition to IL-33, the roles of epithelial cell-derived type 2-initiating cytokines TSLP and IL-25 have also been investigated. Several studies using animal conjunctivitis models have revealed that epithelial cell-derived TSLP plays important roles in the initiation of innate immunity-driven type 2 immune responses induced by SRW pollen [54,55] and papain [51]. In contrast, no significant differences in clinical symptoms and conjunctival inflammation were found between IL-25 knockout and wild-type mice in ragweed-induced experimental allergic conjunctivitis [11] and papain-induced innate immunity-driven type 2 immune responses [51]. As a result, IL-25 may play a smaller role in conjunctival inflammation than other cytokines.

#### 3.3.5. Role of IL-1α in Mouse Mite-Induced AKC Model

Shiraki et al. demonstrated a mouse model of AKC induced by crude house-dust-mite antigen [56]; this mouse model demonstrated clinical manifestations similar to those of patients with AKC. In this model, mice challenged with high-dose house-dust-mite antigen exhibited ulcer-like lesions in the cornea, which showed epithelial damage or defects with severe eosinophilic infiltration histologically. Subcutaneous injection of anti-IL-1α antibody to the eyelids attenuated eosinophilic inflammation in this model. Topical administration of IL-1 receptor antagonists also reportedly suppresses allergic conjunctivitis induced by cat dander in mice [57]. Considering the increase of IL-1α on the ocular surface in patients with AKC [58,59], alarmin IL-1α could also be a major exacerbating factor in ocular allergy.

### 3.4. The Expression of Alarmins in Patients with Ocular Allergy

IL-33 is reportedly highly expressed in epithelial cells and vascular endothelial cells in conjunctival tissues of VKC/AKC [12]. In addition, IL-33 levels in the tears of patients with primary Sjogren’s syndrome were significantly increased and correlated with the severity of ocular surface epithelial damage as well as IL-4 and IL-5 levels in the tears [60]. Epithelial IL-33 may also play a role in dry eye in addition to ocular allergy.

The expression of TSLP in conjunctiva and tear fluid has been shown in several studies. The expression of TSLP mRNA and protein was observed in the conjunctivae of patients with VKC/AKC, but not in control subjects [61,62]. Immunohistochemical study showed preferential expression of TSLP in the epithelial cells of the giant papillae [61]. Zheng et al. have also investigated the expression of TLSP in conjunctival scrapings and tears of patients with VKC, seasonal allergic conjunctivitis (SAC), and perennial allergic conjunctivitis (PAC) [63]. The expression of TSLP in conjunctivae and tears was increased in patients with not only VKC but also SAC and PAC compared to controls. Moreover, tear TSLP concentrations and conjunctival epithelium mRNA levels in the VKC group were significantly higher than in the SAC and PAC groups.

Considering IL-1α, the expression of IL-1α mRNA on the ocular surface [58] and serum levels of IL-1α [59] are significantly increased in patients with VKC, and cultured fibroblasts from patients with VKC show an increased synthesis of IL-1α [59].

HMGB1 was also found to be significantly increased in tear fluids and serum in patients with VKC [64,65]. A significant reduction in serum HMGB1 levels was also detected following treatment with cyclosporine eye drops [64]. Although the precise mechanisms remain unknown, alarmins may also play a role in the systemic inflammation in VKC [59,65].

While the involvement of alarmins in ocular allergic diseases is established, the expression and roles of alarmins may vary depending on the phase of inflammation. Studies regarding kinetic and pre- and post-treatment alarmin expression are currently limited. As a result, further research is needed to better understand the expression and roles of alarmins in patients with these conditions.

## 4. Conclusions

The corneal epithelium has a potent barrier function under normal conditions, preventing the entry of various inflammatory mediators from tear fluid into the corneal stroma. During severe allergic inflammation in the conjunctiva, corneal epithelial cells are damaged and lose their barrier function owing to alarmins, cytotoxic proteins from eosinophils, type 2 cytokines, and mechanical stress caused by scratching of the eyes. Alarmins from the damaged epithelium or eosinophils subsequently infiltrate the corneal stroma and activate corneal fibroblasts to exacerbate eosinophil infiltration. Once corneal and conjunctival epithelial cells are damaged, a vicious loop of corneal injury and exacerbation of allergic inflammation in the conjunctiva begins. In severe types of ocular allergic disease, such as AKC/VKC, alarmins and maintenance of a healthy corneal epithelial barrier should be important future therapeutic targets.

In recent years, monoclonal antibody therapies have been used to treat various allergic diseases [66,67]. Although there is no clinical application of biologics for ocular allergic diseases at this time, cases of AKC successfully treated with the anti-IL-4 receptor α antibody, dupilumab, have been reported [68]. Many antibodies against alarmins, such as anti-IL-33 antibodies, have been developed and are currently undergoing clinical trials for asthma, chronic rhinosinusitis, and atopic dermatitis [69,70]. These antibodies against alarmins are expected to be used to treat refractory severe ocular allergic diseases in the near future.

## Data Availability

Not applicable.
